# LONGITUDINAL CHANGES IN WELL-BEING ASSOCIATED WITH AGING ATTITUDE, COGNITIVE STATUS, AND WIDOWHOOD TRANSITION

**DOI:** 10.1093/geroni/igad104.1667

**Published:** 2023-12-21

**Authors:** Lydia Li

## Abstract

This symposium brings together four studies that employ national data collected from older Americans to examine longitudinal changes in older adults’ cognitive, social, and psychological health. The first study focuses on the role of self-perception of aging in memory decline and social mechanisms linking aging perception and memory. It found that negative perceptions of aging predicted memory decline 8 years later and that loneliness and social isolation were pathways. The second study is concerned with neuropsychological changes before the onset of dementia. Using latent growth modeling, it found that the trajectory of depression increased over time in a quadratic fashion before the onset of incident dementia. Further, there were racial/ethnic group differences in levels of depression. Using fixed-effect linear regression, the third study reports that changes in cognitive status were associated with changes in social isolation in older adults. Specifically, it found that transitioning from cognitive intact to cognitive impairment status was associated with increased social isolation. The fourth and final paper examines changes in psychological well-being related to the transition to widowhood. It found that during the year right after the spouse died, older adults experienced increased depressive symptoms and reduced positive well-being. But adaptation is evident—the surviving spouse returned to pre-loss levels of psychological well-being around two years after the death. Together, the four studies show that attitude, cognitive status, and life course transition shape the dynamics of cognitive, social, and psychological health in later life.

